# Quenching for Microalgal Metabolomics: A Case Study on the Unicellular Eukaryotic Green Alga *Chlamydomonas reinhardtii*

**DOI:** 10.3390/metabo8040072

**Published:** 2018-10-31

**Authors:** Rahul Vijay Kapoore, Seetharaman Vaidyanathan

**Affiliations:** 1 Department of Chemical and Biological Engineering, ChELSI Institute, Advanced Biomanufacturing Centre, The University of Sheffield, Sheffield S1 3JD, UK; S.Vaidyanathan@Sheffield.ac.uk; 2 Department of Biosciences, College of Science, Swansea University, Swansea SA2 8PP, UK

**Keywords:** metabolomics, microalgae, quenching, *Chlamydomonas reinhardtii*, gas chromatography mass spectrometry (GC-MS)

## Abstract

Capturing a valid snapshot of the metabolome requires rapid quenching of enzyme activities. This is a crucial step in order to halt the constant flux of metabolism and high turnover rate of metabolites. Quenching with cold aqueous methanol is treated as a gold standard so far, however, reliability of metabolomics data obtained is in question due to potential problems connected to leakage of intracellular metabolites. Therefore, we investigated the influence of various parameters such as quenching solvents, methanol concentration, inclusion of buffer additives, quenching time and solvent to sample ratio on intracellular metabolite leakage from *Chlamydomonas reinhardtii*. We measured the recovery of twelve metabolite classes using gas chromatography mass spectrometry (GC-MS) in all possible fractions and established mass balance to trace the fate of metabolites during quenching treatments. Our data demonstrate significant loss of intracellular metabolites with the use of the conventional 60% methanol, and that an increase in methanol concentration or quenching time also resulted in higher leakage. Inclusion of various buffer additives showed 70 mM HEPES (4-(2-hydroxyethyl)-1-piperazineethanesulfonic acid) to be suitable. In summary, we recommend quenching with 60% aqueous methanol supplemented with 70 mM HEPES (−40 °C) at 1:1 sample to quenching solvent ratio, as it resulted in higher recoveries for intracellular metabolites with subsequent reduction in the metabolite leakage for all metabolite classes.

## 1. Introduction

The major bottlenecks associated with sample preparation include efficient sampling, quenching, and extraction of metabolites, achieved with minimal alteration of the internal metabolome signature. In order to retain a valid snapshot of the metabolome, rapid sampling and quenching of enzyme activities is a crucial step in any metabolomics workflow. Ideally, quenching solvent should halt the constant flux of metabolism and high turnover rate of metabolites without causing any damage to the cell membrane/wall thereby avoiding any leakage of intracellular metabolites [1,2]. Quenching with 60% *v*/*v* cold methanol at −40 °C or −50 °C has been used widely in the past for microbial, fungi, yeast and plant metabolomics. However, potential problems connected to the leakage of intracellular metabolites with cold methanol quenching was reported later for yeast [3] and bacterial cells [4,5,6,7]. Various alternatives to cold methanol quenching, such as filter culture methodology [8], fast filtration [4], mass balance approach [9] and use of alternative quenching solvents (such as glycerol-saline, methanol/glycerol and methanol/NaCl) have been evaluated for bacterial metabolomics [5,10,11]. However, all suggested alternatives have advantages and disadvantages, and more importantly cannot be directly applied to a given organism, without prior evaluation [12]. In addition, these alternatives have also been shown to add difficulties in the overall metabolomics workflow. For example, the higher viscosity and hygroscopicity of glycerol has been shown to result in prolonged processing time (during separation of glycerol from cells) [13] and interference with the commonly employed silylation derivatization reaction (required for gas chromatography mass spectrometry (GC-MS) analysis) [14,15]. 

To date, the most widely used quenching method is that of 60% aqueous methanol, which usually results in leakage of the intracellular metabolites, and requires an accurate balancing of the quenching supernatant and sample [16]. Sampling methods optimised for prokaryotes cannot be simply adopted for eukaryotic cells due to basic differences in the cell structure. The inclusion of additives to methanol will act as a buffer or will restrict osmotic shock, leading to a decrease in leakage, as has been reported with bacterial cells [17], yeast [9,14] and mammalian cells [18,19]. The inclusion of tricine buffer in 60% aqueous methanol has been shown to result in lower recoveries for the intracellular metabolites with GC-MS because of the interference of tricine with derivatization reactions [20]. However, with other additives within the same biological system, contradictory conclusions have been reported (Table S1). A few reports have covered the influence of methanol concentration on metabolite leakage [9,21], however, even in these the approach has been kept limited to the quantification of specific metabolites. Another important factor that needs to be evaluated includes the influence of initial (before quenching) and the final temperature of methanol (sample-quenching solvent mixture). The lower the temperature, the slower the turnover rate of all the enzymes within the cell will be and hence more efficient is the quenching process [19]. However, only Canelas and co-workers [9] appear to have evaluated this factor for yeast, where the authors concluded that “leakage free quenching” can be achieved with the use of pure methanol rather than 60% methanol with quenching solvent to sample ratio of 5:1, and at −40 °C or lower. However, with a similar “leakage free quenching” method, Kim and co-workers [22] have demonstrated severe leakage in *Saccharomyces cerevisiae*, suggesting reduced membrane integrity caused by the use of extreme cold quenching conditions. The influence of contact time of sample with the quenching solvent has not been investigated yet, where higher leakage of specific classes of metabolites is likely to occur upon increasing the exposure time of sample to that of quenching solvent [4]. Finally, the influence of sample to quenching solvent ratio should also be carefully considered, as it directly alters the temperature of the quenching process and might help in minimising intracellular metabolite leakage. Schädel and co-workers [13] evaluated this factor for *Escherichia coli* metabolomics and observed no change in the impact of methanol with the temperature variation.

As discussed above, despite contradicting reports, quenching protocols have been rigorously studied and optimised for yeast, mammalian and bacterial models. Lee and Fiehn suggested yeast can be regarded as a good proxy for *Chlamydomonas reinhardtii* as both are eukaryotes and have sturdy cell walls compared to that of bacterial models which are easily prone to metabolite leakage caused by harsh quenching treatments [23]. However, it is important to note that minor differences in cellular characteristics including membrane, wall structure, size and sampling techniques employed, can influence the efficiency of quenching, recovery of different metabolite classes and the rate of metabolite leakage. Therefore, optimised quenching methods for yeast, mammalian and bacterial models cannot be simply adopted for microalga without critically evaluating them. In case of microalgal samples, we are not aware of many reports to date, on this issue. Lee and Fiehn [23], reported quenching cultures of *C. reinhardtii* with 70% aqueous methanol (−70 °C) with 1:1 ratio to sample, which reportedly resulted in minimum leakage of intracellular metabolites. In this case, the resultant final concentration of methanol was 35% and final temperature recorded was −20 °C. This finding was in contrast to their previously published report, where no leakage was reported and results concluded that *C. reinhardtii* cultures are resistant to quenching with cold methanol [24]. 

The objective of this investigation is to examine quantitatively, the influence of the above mentioned factors on the extent of metabolite leakage in *C. reinhardtii* cultures. To achieve our objective, we have designed and categorised the experiments into three different approaches. Approach 1 involves evaluation of selected quenching solvents (nine in total) with varying methanol concentration and with various buffer additives, on the extent of metabolite leakage. Approach 2 investigates the effect of prolonged quenching duration on metabolite leakage and approach 3 investigates the effect of the ratio of quenching solvent to culture on metabolite leakage. Furthermore, we investigated the recovery of twelve different metabolite classes using the GC-MS technique across different quenching treatments. The evaluation was based on recovery of a median number of metabolites within each class in all possible sample fractions and recovery of average metabolite peak intensity per class (derived from normalised median peak intensities of metabolites within each class). 

## 2. Results and Discussion

**Figure 1:**
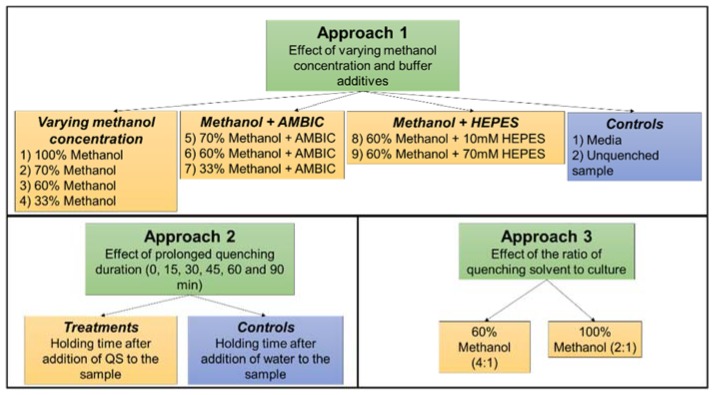
Experimental design and general workflow adopted for the evaluation and optimisation of quenching protocols for *Chlamydomonas reinhardtii*.

To achieve our objectives in a broader sense, we have designed the experiments and categorised them into three different approaches as illustrated in Figure 1. In all the applied quenching treatments, the temperature during the quenching process was maintained below −20 °C. Although metabolites with high turnover rates such as adenosine triphosphate (ATP) and adenosine diphosphate (ADP) might still remain active at −20 °C, they are not usually detected with GC-MS based analysis. In most of the studies that evaluated the quenching methods for bacterial models [13,17], conclusions were drawn primarily based on ATP assay and adenylate energy charge. However, comment on quenching efficiency protocols cannot be made only based on these assays as it does not take into consideration the possible alterations in the rest of the metabolome. In the present study, we focussed primarily on GC-MS based analysis and the optimisation of quenching protocols were done by taking into consideration quantification of detectable compounds with GC-MS analysis. In all the three approaches, evaluation and comparison within different treatments were based on two response variables, where only features that were present in at least three biological replicates out of five were considered for further analysis:

- Response variable 1: Median number of metabolites recovered in;
  (a) Cell extracts
  (b) In quenching supernatant
  (c) Only in cell extract and not in supernatant (unique to cells)
  (d) Only in supernatant and not in cell extract (unique to supernatant) and
  (e) In both the cell extracts and supernatants (common to both)
- Response variable 2: Recoveries of metabolites within twelve different classes of metabolites with respect to their normalised median peak intensities, and represented by the average metabolite peak intensity for each class.

We analysed the unquenched samples in order to get an estimate of extracellular metabolites accurately, as an unquenched sample serves as a reliable standard for comparison of the extracellular metabolome data from the quenched sample. In addition, we have also analysed the culture media as a control for appropriate quantification of an intracellular pool, to account for the leftover media components, if any. 

### 2.1. Leakage Based on Recovery of Median Metabolite Numbers

#### 2.1.1. Effect of Methanol Concentration and Inclusion of Buffer Additives on Metabolite Leakage (Approach 1) 

**Figure 2:**
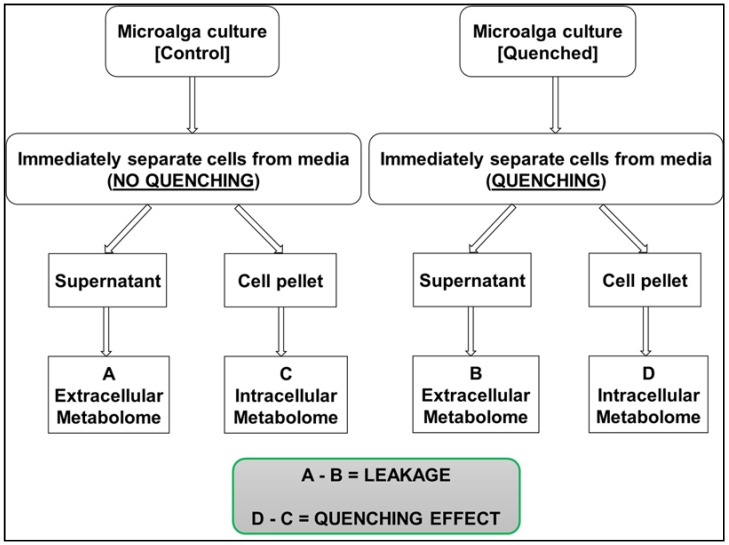
Schematic displaying the necessary correction required for appropriate calculation of intracellular metabolites after monitoring cell-free supernatant of quenched, non-quenched cells and the blank medium.

In order to compare the extent of metabolite leakage and recoveries of intracellular metabolites under different methanol concentrations and additives, cells of *C. reinhardtii* were harvested and quenched as described in the experimental section with nine different quenching solvents and additives. Variation in quenching treatments was tested to investigate the different effects and the results are summarised in Table 1. For an overall comparison, the harvested cell extracts, a cell-free supernatant of both quenched and non-quenched cells along with the blank sample (culture medium) were analysed to determine the extent of leakage of intracellular metabolites during quenching. After monitoring cell-free supernatant of quenched, non-quenched cells and the blank medium, the necessary correction was done for appropriate calculation of intracellular metabolites as shown in Figure 2. 

**Figure 3:**
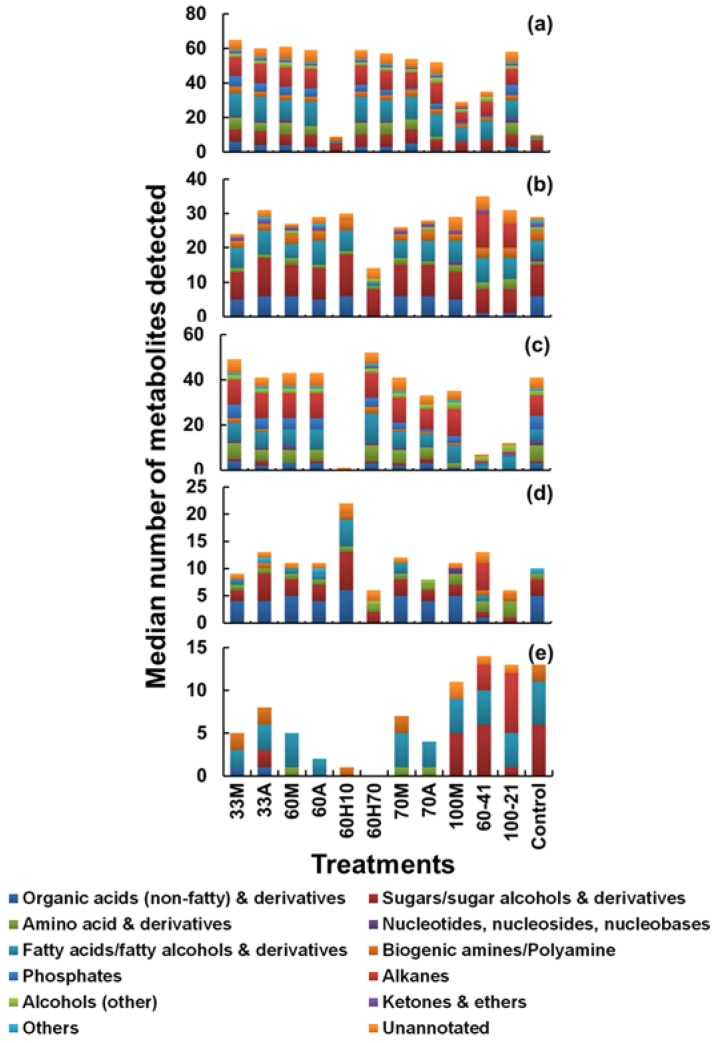
A summary of the unique recovery efficiency of all the nine quenching treatments involved in Approach 1 and 3. The X-axis represents different sampling treatments: 33M = 33% methanol; 33A = 33% methanol + ammonium bicarbonate (AMBIC); 60M = 60% methanol; 60A = 60% methanol + AMBIC; 60H10 = 60% methanol + 10 mM 4-(2-hydroxyethyl)-1-piperazineethanesulfonic acid (HEPES); 60H70 = 60% methanol + 70 mM HEPES; 70M = 70% methanol; 70A = 70% methanol + AMBIC; 100M = 100% methanol; 60–41 = 60% methanol with solvent to sample ratio 4:1 and 100–21 = 100% methanol with solvent to sample ratio 2:1. After all the treatments, the extracted metabolites from cell extracts, cell-free supernatant post quenching and blank samples were analysed by gas chromatography mass spectrometry (GC-MS). (**a**) Metabolites identified in cell extracts only, (**b**) metabolites identified in supernatant only, (**c**) metabolites present only in cell extract (and not in the supernatant)—unique to cells, (**d**) metabolites present only in supernatants (and not in cell extract)—unique to supernatants, (**e**) metabolites present in both the cell extract and supernatant—(common to both).

A summary of the unique recovery efficiency of all the nine quenching treatments is shown in Figure 3, where the metabolite class and numbers detected for various treatments are plotted. Figure 3a shows the number of metabolites detected in the cell pellets, Figure 3b those detected in the cell-free supernatant, Figure 3c indicates the number of metabolites present in the cells but not in the supernatants, Figure 3d indicates the number of metabolites present in the supernatants and not in the cells, and Figure 3e indicates the number of metabolites present in both the cell pellet and the supernatant. Higher metabolite numbers in the supernatant (Figure 3b), relatively high numbers detected in both the cells and the supernatants (Figure 3e), and corresponding low numbers unique to the cell pellets (Figure 3c) indicate high metabolite leakage. A higher proportion of metabolites detected in Figure 3e, compared to that detected in Figure 3c or Figure 3d indicates that there is an increased chance of metabolite leakage. 

As can be seen from Figure 3c, 60% aqueous methanol supplemented with 70 mM HEPES (60H70) yielded higher recoveries of metabolites unique to cells as compared to other treatments. Correspondingly, metabolites common to both the cells and supernatant (Figure 3e) are the lowest. The number of metabolites detected in the supernatant is also the least compared to other treatments (Figure 3d). Therefore, among all the treatments, 60H70 seems to preserve the integrity of *C. reinhardtii* cells, resulting in higher recoveries of intracellular metabolites with minimal leakage into the extracellular environment. 

Among other treatments, we did not observe any variations between 60M and 60A treatments. On the other hand, recovery of intracellular metabolites decreases with corresponding increase in extracellular levels as the methanol concentration increases. As can be seen from Figure 3c, higher recovery of intracellular metabolites was observed with 33M compared to 60M, 70M and 100M whereas metabolites common to both the cells and supernatants (Figure 3e) are lower with 33M compared to other treatments. Among treatments where methanol was supplemented with 0.85% AMBIC, 60A yielded higher recoveries of metabolites unique to cells (Figure 3c) compared to 33A and 70A. Correspondingly, metabolites common to both the cells and supernatants (Figure 3e) are the lowest. However, as mentioned above, we did not observe any major change in recoveries between 60M alone compared to that of 60A, therefore the use of 60M alone would be advantageous over the use of methanol supplemented with AMBIC. Moreover, comparison within treatments where non-buffered methanol was used for quenching, quenching with 33M seems to be a better option among non-buffered methanol and buffered with AMBIC. Therefore, in studies involving use of MS based hyphenated techniques (especially liquid chromatography mass spectrometry (LC-MS)), where use of salts/buffers as quenching additives introduces an additional source of variance to the experimental procedure, increases complexity of data and causes ion suppression in LC-MS, we strongly recommend use of 33M as a second alternative to 60H70. However as demonstrated earlier [25], an addition of HEPES has no apparent interference with the derivatization reactions and GC-MS analysis. 

In summary, recovery of intracellular metabolites was noticed to decrease with corresponding increase in extracellular levels, as the methanol concentration in the quenching solvent increased. Our results were in agreement with that of previous reports [7,21] and in contrast to reports [9,13] where authors reported higher recovery of intracellular metabolites from *E. coli* and *S. cerevisiae* respectively with the corresponding increase in methanol concentration. In contrast, Tredwell and co-workers [20] reported that no major difference in leakage from *Pichia pastoris* was observed with the varying concentration of methanol or the inclusion of various buffer additives in quenching solvent. Furthermore, Canelas and co-workers [9] tested the influence of buffer additives (HEPES, AMBIC and/or tricine) in methanol on metabolite leakage from yeast, and reported no significant benefit in buffering or increasing the ionic strength of the quenching solvent. The authors reported slightly lower intracellular recoveries, which is completely contradictory to our finding with *C. reinhardtii*. The possible reasons behind this contradiction might be differences in the organism/sample type. Moreover, it is important to note that conclusions were drawn from an estimation of only two specific metabolites which were being used as representative examples for the respective class. Similarly, in contrast to our findings where 60H70 yielded highest intracellular recoveries with minimal leakage, Schädel and co-workers [13] reported higher leakage in *E. coli* with the inclusion of HEPES to methanol compared to conventional 60% methanol. These contradictory observations further support our previous conclusions [1], that sampling and quenching techniques are highly sample/cell dependent and needs critical evaluation and validation for every organism under investigation before being adopted for a quantitative metabolomics study.

#### 2.1.2. Effect of Prolonged Exposure to Quenching Solvent on Metabolite Leakage (Approach 2)

**Figure 4:**
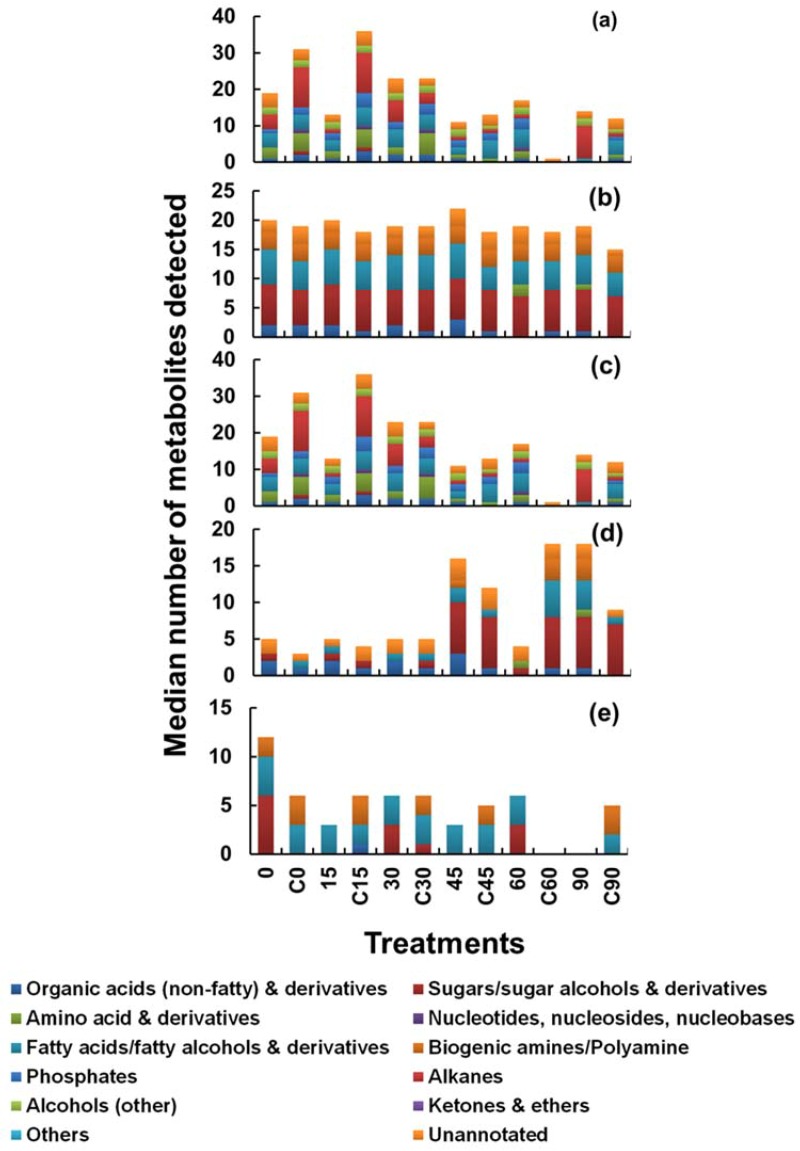
A summary of the unique recovery efficiency of all the six quenching treatments involved in Approach 2. The X-axis represents different time intervals along with their control samples (C), for each time interval. After all treatments the extracted metabolites from cell extracts, cell-free supernatant post quenching and blank samples were analysed by GC-MS. (**a**) Metabolites identified in cell extracts only, (**b**) metabolites identified in supernatant only, (**c**) metabolites present only in cell extract (and not in the supernatant)—unique to cells, (**d**) metabolites present only in supernatants (and not in cell extract)—unique to supernatants, (**e**) metabolites present in both the cell extract and supernatant—(common to both).

Ideally, quenched cells should be processed as quickly as possible in order to avoid the leakage of intracellular metabolites into the extracellular environment, as prolonged contact time of cells with the quenching solvents might increase the chances of metabolite losses via diffusion of small metabolites through the cell membrane. In 1992, De Koning and Van Dam [26] demonstrated no leakage of intracellular metabolites from yeast sample after exposure of quenching solvent to sample for 30 min. This has been evaluated in the past for *S. cerevisiae* [9] and for the fungus *Penicillium chrysogenum* [21], however, there are no reports of such studies carried out on microalgal samples. Therefore, to test this theory for microalga, cells of *C. reinhardtii* were exposed to the quenching solvent (60% *v*/*v* aqueous methanol) for a prolonged period of various time intervals to evaluate the extent of metabolite leakage. Briefly, 1 mL of cell suspension was rapidly plunged into 1 mL of quenching solvent as described in the experimental section. To evaluate the extent of metabolite leakage in response to prolonged exposure to the quenching solvent, a broader range of time intervals were selected including 0, 15, 30, 45, 60 and 90 min. In the case of 0 min treatment, samples were processed immediately by centrifugation, whereas for other treatments prolonged exposure was achieved by leaving the quenched samples at −40 °C for the above specified time intervals prior to centrifugation. A parallel set of samples were processed as a control for each time interval. For example, in the case of the control sample for the 15 min time interval, 1 mL of cell suspension was harvested and allowed to stand for 15 min followed by addition of 1mL of water and subsequently centrifuged. The addition of water was done in order to account for the variations caused by dilution. Water was purposely selected in this case instead of any other buffer solution such as phosphate buffer saline (PBS), as the addition of PBS might result in an extremely higher concentration of phosphates and will interfere in subsequent GC-MS analysis or will result in overestimation of phosphates in intracellular pools. For an overall comparison, the harvested cell extracts, a cell-free supernatant of both quenched (samples) and non-quenched cells (controls) for all the above specified time intervals along with the blank sample (culture medium) were analysed to determine the extent of metabolite leakage in response to prolonged exposure to the quenching solvent. After monitoring cell-free supernatant of both quenched, non-quenched cells and the blank sample, the necessary correction was done for appropriate calculation of intracellular metabolites as shown in Figure 2. A summary of the unique recovery efficiency of all the applied treatments within this approach is shown in Figure 4, where the metabolite class and median numbers detected for various treatments are plotted. 

As can be seen from Figure 4c, samples processed at the 0 time interval yielded higher recoveries of metabolites compared to all other treatments except for the 30 min interval where surprisingly higher numbers of metabolites were detected than that of the 0 min time interval. A similar trend was observed with the control samples where higher recoveries were observed with C0 treatment compared to other treatments except for C15 where again surprisingly higher recoveries were observed than that of C0. The possible reason behind higher recoveries might be degradation or inter-conversion of metabolites. Correspondingly higher number of metabolites unique to the supernatant (Figure 4d) were detected as the contact time of cell suspension was prolonged for more than 30 min with that of quenching solvent. Approximately four-fold increase in recoveries of extracellular metabolite numbers was observed with 45, 60 and 90 min treatments compared to that of the 0 min treatment (Figure 4d). No variations in recoveries of metabolites classes and numbers were observed between 0, 15 and 30 min time intervals whereas a small increase in the recoveries of metabolites was observed with control treatments as contact time was increased from 0 to 15 and then to 30 min indicating increased metabolite leakage. 

#### 2.1.3. Effect of Quenching Solvent to Culture Ratio (Temperature Influence) (Approach 3)

Influence of sample to quenching solvent ratio was studied in the past for *S. cerevisiae* and *E. coli*, where quenching with pure methanol at sample to solvent ratio of 1:5 at −40 °C has been shown to have reduced leakage of intracellular metabolites compared to use of 60M. In order to test this parameter with microalgal samples and to study the influence of quenching solvent and sample mixture, temperature and final concentration of methanol after quenching, 1 mL culture of *C. reinhardtii* was harvested and rapidly quenched with either 100% methanol with quenching solvent to sample ratio of 2:1 (100–21) or with 60% aqueous methanol with quenching solvent to sample ratio of 4:1 (60–41). Both the quenching solvents were pre-chilled to −50 °C prior to quenching. Addition of the cells to the quenching solution increased the temperature by no more than 15 °C, thereby keeping the resulting mixture temperature below −20 °C sufficient to stop the metabolism as demonstrated in past [13,16,27]. For an overall comparison, the harvested cell extracts, a cell-free supernatant of both quenched and non-quenched cells along with the blank sample (culture medium) were analysed and the necessary correction was done for appropriate calculation of intracellular metabolites and to determine the extent of leakage of intracellular metabolites during quenching. The results of the investigation are summarised in Figure 3.

With both the treatments 60–41 and 100–21 higher metabolite numbers in the supernatant (Figure 3b), relatively high numbers detected in both the cells and the supernatants (Figure 3e), and corresponding low numbers unique to the cell pellets (Figure 3c) were observed, suggesting high metabolite leakage. A higher proportion of metabolites detected in Figure 3e, compared to that detected in Figure 3c or Figure 3d confirms that there is an increased chance of metabolite leakage with both the treatments. With this approach, we did not observe any significant improvements in preserving the cell integrity by altering the final methanol concentration in the resultant mixture after quenching. Moreover, an increment in quenching solvent to sample ratio in order to keep the temperatures of the resulting mixture below −20 °C for the effective halting of metabolism seems to have no influence on preserving the cell integrity and in minimising the metabolite leakage. In contrast, higher leakage was observed with this approach compared to approach 1.

### 2.2. Leakage Based on Metabolite Peak Intensities

With respect to analysis of metabolite classes recovered and the median intensities of individual metabolites identified within each class, in total, 151 unique putative metabolite identifications were made in approach 1 and 3, and 123 made in approach 2, across all treatments. In addition, there were unique features that did not match with metabolites on the database and were hence classed as “unannotated”. These were 46 in approach 1 and 3, and 42 in approach 2 (Tables S2 and S3).

**Figure 5:**
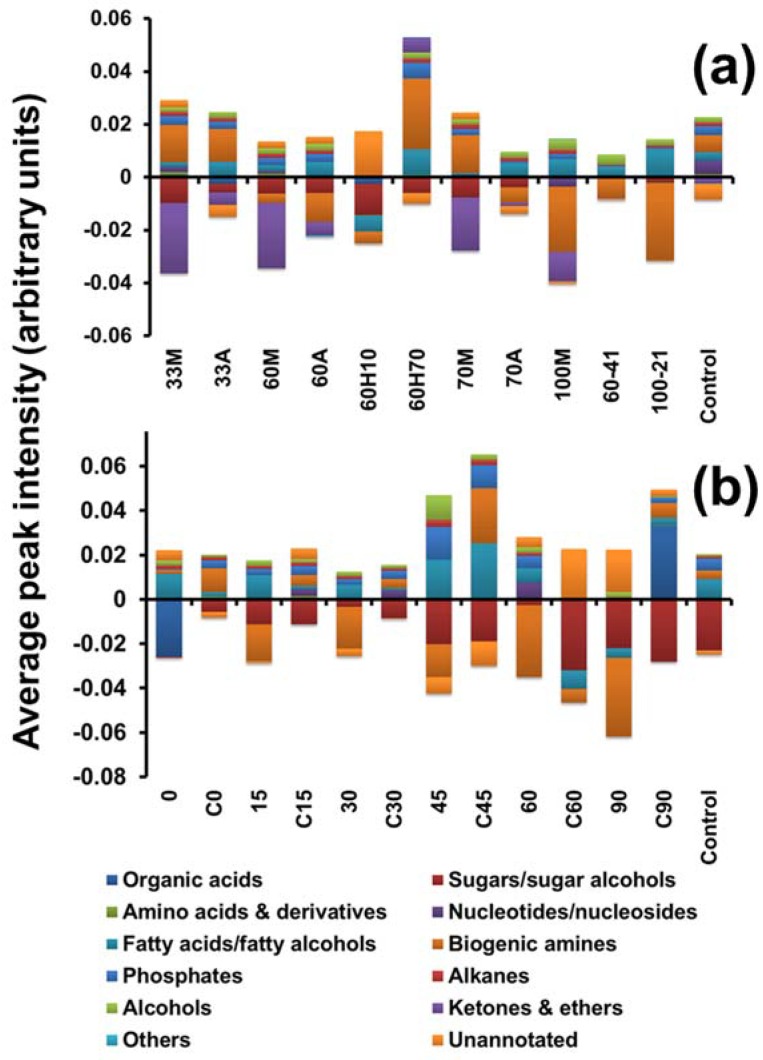
Difference in the average peak intensities of metabolites between cell extracts and supernatants, within each class is plotted for each treatment in approach 1 and 3 (**a**) and approach 2 (**b**).

The results are compiled in Figure 5, where the median peak intensities of metabolites were averaged within each class and plotted for the different treatments in the three approaches, 1–3. Values above zero indicate higher detection in cell extracts than supernatants and values lower than zero indicate higher detection in supernatant than in cell extracts, suggesting leakage. For approach 1, superior recoveries of organic acids were observed with the 33M treatment, with minimal leakage. With sugars/sugar alcohols and derivatives, extracellular levels were higher for all treatments, except 100M. Since the observation is made with almost all conditions, it is possible that this is a result of leakage metabolism, rather than leakage due to quenching. Higher recoveries of amino acids were observed in the cell extracts with 60M and 33M treatments, suggesting minimal leakage of this class in these treatments. A higher leakage of amino acids was observed with 100M and 60H10 treatments. This is in agreement with the observation of Britten and McClure [28], where the authors demonstrated leakage of all free amino acids in *E. coli* upon variation in the osmolality of the surrounding medium. Nucleotides/nucleosides/nucleobases showed poor recoveries. The possible reason for lower recoveries in both the cell extracts and the supernatant might be that GC-MS is not an ideal technique for analysing this class. This has been previously suggested in a few studies. In such cases, LC-MS or capillary electrophoresis mass spectrometry (CE-MS) [29,30] might be a suitable alternative to GC-MS in order to improve the metabolome coverage with respect to nucleotides/nucleosides/nucleobases. Our results were in agreement with the results reported elsewhere [3,6] where no detectable leakage of nucleotides was observed with the cold methanol solution in the case of the yeast sample (which in our case corresponds to the 60M treatment). Fatty acids/fatty alcohols and derivatives showed minimal leakage in most treatments, and in particular in 100M and 60H70 treatments. Somewhat similar conclusions were reported in a previously published report [15], where higher peak intensities were reported for fatty acids with glycerol-saline quenching solution and extraction with 100% methanol using freeze-thaw cycles. Biogenic amines, in particular, ethanolamine, putrescine and tryptamine, showed minimal leakage in the 60H70 treatment but were also retained better in 33M, 33A and 70M treatments (Figure 5). Phosphates and alkanes were recovered in the cell extract with minimal leakage into supernatant with all the treatments except with 60H10 treatment, where negligible amounts of phosphates and alkanes were recovered in both the cell extract and the supernatant. Among all the treatments, higher recoveries were observed with 60H70. A gradual increase in the methanol concentration of quenching solvent resulted in a gradual decrease in the corresponding intracellular levels of phosphates which was in further agreement with a previously published report [9]. Alcohols were retained better in all treatments except 60H10. In the case of ketones and ethers, all the treatments (except with 60H70) showed leakage (Figure 5a).

For approach 2, total median intensities of all organic acids showed gradual decrease in cell extract recoveries from 15 min onwards up to 45 min hold time with corresponding increased recoveries in supernatant indicating leakage, whereas minor recoveries were observed in cell extracts with 45, 60 and 90 min intervals suggesting complete degradation or inter-conversion of all organic acids. In the case of sugars/sugar alcohols and derivatives lower recoveries for intracellular levels were observed as the exposure of cell suspension to the quenching solvent was increased from 0 to 30 minutes, indicating time dependant slow release of sugars into the extracellular medium, with complete leakage into the medium at 45 and 90 min exposures (Figure 5b). In the case of amino acids and derivatives, no leakage was observed up to 30 min exposure. Our findings were in agreement with a previously published report [6] where leakage of amino acids was increased with the increase in a contact time of sample with the quenching solvent. A gradual increase in extracellular levels of fatty acids/fatty alcohols and derivatives were observed as the hold time increased from 0 to 45 min (0-SN, 15-SN, 30-SN and 45-SN) indicating slower leakage of these less polar or semi-polar metabolites via diffusion. Putrescine and other biogenic amines were detected with all the applied quenching treatments. The results suggest slower time dependant leakage of all biogenic amines/polyamines, as extracellular levels were gradually increased with the corresponding decrease in the intracellular levels as the exposure to quenching solvent was increased from 0 min to 45 min. In the case of phosphates, absolutely no recoveries were observed in the quenching supernatant among all the applied treatments. A possible reason might be the larger and more polar nature of these metabolites making their diffusion through the cell wall difficult as suggested elsewhere in the case of yeast and fungi samples [9,21], leading to less leakage compared to other classes such as amino acid and organic acids which are smaller compounds and might be released more easily than the larger ones. However, despite their non-polar nature, similar trends as that of phosphates were observed with alkanes where no recoveries were observed in the quenching supernatant. The possible reason might be their larger size making their diffusion through the cell wall difficult as most of the alkanes that were recovered have molecular weight (MW) ranging from 212 to 352. A similar trend was observed with alcohols, ketones and ethers. In summary, nearly all the metabolite classes showed a gradual decrease in the intracellular levels with a corresponding increase in the extracellular levels, as the contact time of sample to quenching solvent was increased. Our findings were in agreement with previously published reports, where similar conclusions were drawn with yeast and bacterial models [6,9,21]. 

For approach 3, in the case of amino acids, organic acids, biogenic amine/polyamines, sugars/sugar alcohols and ketones and ethers very low recoveries were obtained in both the cell extracts and in the supernatant with both the quenching treatments compared to those in approach 1. With both the treatments, no recoveries were observed for nucleotides/nucleosides/nucleobases and other classes of metabolites. Superior recoveries of fatty acids/fatty alcohols and derivatives were obtained in the cell extract with the 100–21 treatment with the corresponding decrease in extracellular levels indicating minimal leakage (Figure 5a). Moreover, the average peak intensities recovered for this class was superior compared to all the applied treatments in approach 1. A similar trend was observed in recoveries of alcohols with 100–21 treatment.

## 3. Materials and Methods 

### 3.1. Chemicals and Analytical Reagents

All chemicals and reagents were obtained from Sigma-Aldrich (Dorset, UK) unless stated otherwise.

### 3.2. Microalgal Cultivation

The *C. reinhardtii* strain (CC4323) was grown until the end of exponential phase (72 h) under constant illumination in a Sanyo incubator (MLR-351H, Sanyo versatile environmental test chamber, Osaka, Japan) at 25 °C in 1 L shake flasks, containing 1 L of TAP medium, under constant illumination at 85 µmol/m^2^/s. The medium (per L) is composed of 2.42 g Tris; 25mL of TAP salts (15 g NH_4_Cl, 4 g MgSO_4_·7H_2_O, 2 g CaCl_2_·2H_2_O in 1 L dH_2_O); 0.375 mL of phosphate solution (28.8 g K_2_HPO_4_, 14.4 g KH_2_PO_4_ in 100 mL dH_2_O); 1mL solution Hunter’s trace elements purchase from the Chlamydomonas Resource Centre (St. Paul, MN, USA) and 1 mL of glacial acetic acid. Cells were harvested at an OD of 1.2 at 680 nm wavelength.

### 3.3. Sampling and Quenching 

To evaluate and minimise the leakage of metabolites during quenching treatments, the overall design of quenching experiments has been categorised into three different approaches as summarised in Figure 1. At the incubation site, 1 mL of cell suspension was rapidly plunged into a 2 mL pre-chilled Eppendorf containing 1 mL of pre-chilled quenching solvent (−50 °C) unless stated otherwise. Addition of the cells to the quenching solvent increased the temperature by no more than 15 °C. The centrifuge was set at −9 °C and the rotor was pre-chilled at −24 °C. The quenched biomass was then centrifuged for 2 min at 2500× *g* at −9 °C. The supernatant was removed rapidly, and transferred to a 2 mL pre-chilled Eppendorf to assess the leakage of internal metabolites. The pellets and supernatant were snap frozen in liquid nitrogen and stored at −80 °C for further analysis.

### 3.4. Metabolite Extraction

The pellets were lyophilized at −50 °C overnight prior to extraction. Briefly, 500 µL of extraction solvent (methanol:chloroform:water, (M:C:W) (5:2:2)) was added to lyophilized cells, as suggested elsewhere [23,31,32], pre-chilled at −48 °C, along with an equal volume of glass beads (425–600 µm i.d., acid washed, from Sigma). Cells were then disrupted using a Cell disruptor, (Genie, VWR, U.K.), designed to simultaneously agitate and vortex at high speed, thereby resulting in a rapid disruption of cells in a small aliquot of extraction solvent (0.5 to 2 mL). A total of 11 cycles of disruption was performed with 2 min disruption, with an interval of 1 min on ice. The sample was then centrifuged at 13,000× rpm, at −9 °C for 15 min to remove any cell debris. The supernatant was transferred to a new pre-chilled Eppendorf tube (−20 °C) and the remaining cell debris was subjected to re-extraction (5 cycles) with 500 µL of extraction. The combined supernatant was then evaporated to dryness using a vacuum concentrator (Eppendorf 5301 vacuum concentrator, Sigma-Aldrich (Dorset, UK)). The dried extract was then stored overnight at −80 °C for further analysis.

### 3.5. Metabolite Derivatization and GC-MS Analysis 

Metabolite derivatization was performed the next day on a stored dried extract, as suggested elsewhere [23]. Briefly, 30 µL of 20 mg/mL methoxyamine hydrochloride in pyridine was added to the dried extract and samples were shaken (200 rpm) for 80 min at 37 °C to protect the aldehyde and ketone groups. The samples were then derivatized by trimethylsilylation of acidic protons by addition of 45 µL MSTFA (N-methyl-N-trimethylsilyltrifluoroacetamide) and incubating them further in shaking condition (200 rpm) at 40 °C for 80 min. GC-MS analysis, metabolite identification and data analysis were conducted as described elsewhere [1,25,33]. 

## 4. Conclusions

In summary, we have performed a comprehensive investigation of appropriate sampling and quenching methods for *C. reinhardtii* using GC-MS, which involved untargeted quantitative analysis of several intracellular and extracellular metabolites. Our optimised miniaturised quenching method requires only 1 mL of microalgal culture, thereby subsequently reducing the volume of quenching solvent required, suitable for processing larger number of samples within a shorter period of time, ideal for metabolomics investigation where a large number of samples need processing. To date, there is no effective alternative to the cold methanol quenching method, where quenching can only be achieved by sharp temperature drift which usually leads to leakage caused by cold shock phenomenon. Hence, for reliable metabolome analysis, it is essential to measure the metabolite levels in all possible fractions and establish mass balance to trace the fate of metabolites during quenching treatment. Our results clearly showed higher losses of intracellular metabolites with the use of conventional 60% methanol for quenching. Analysis with respect to the influence of varying methanol concentration in quenching solvent on the extent of metabolite leakage clearly showed higher leakage with the increase in methanol concentration. On the other hand, analysis with respect to the inclusion of various buffer additives to quenching solvent showed 60% aqueous methanol supplemented with 70 mM HEPES to recover higher intracellular levels for nearly all metabolite classes with minimal leakage, supporting our previous findings with adherent mammalian cells [25]. An increment in quenching solvent to sample ratio in order to keep the temperatures of the resulting mixture below −20 °C for the effective halting of metabolism seems to have no influence on preserving the cell integrity and in minimising the metabolite leakage. In contrast, higher leakage was observed with this approach. The study of prolonged exposure of a sample to quenching solvent has shown a higher amount of leakage as the contact time of sample to that of quenching solvent increases. Hence for quantitative metabolomics studies in *C. reinhardtii*, we strongly recommend quenching with 60% aqueous methanol supplemented with 70 mM HEPES (−40 °C) at 1:1 sample to quenching solvent ratio, as it resulted in higher recoveries for intracellular metabolites with subsequent reduction in the metabolite leakage for all classes of metabolites. We believe the outcome of this research and the optimised quenching method can be directly applied and extended to other similar freshwater microalgal cultures or indeed cells with a similar cell envelope architecture and provides a standardised approach to metabolomics validations in other organisms.

## Figures and Tables

**Table 1:** Variation in quenching treatment tested to investigate the effects of varying methanol concentration, effect of inclusion of various buffer additives to quenching solution, effect of temperature and effect of sample to quenching solvent ratio on metabolite leakage.

	Method Code	Addition of Buffer	Initial Quenching Solvent Temperature (°C)	Initial Concentration of Methanol (*v*/*v*) (%)	Sample/Quenching Solvent Ratio	Final Concentration of Methanol after Quenching (*v*/*v*) (%)	Final Temperature of Resultant Mixture after Quenching (°C)
**Approach 1**	33M	x	−50	33	1:1	17	−20
	33A	0.85% AMBIC	−50	33	1:1	17	−20
	60M	x	−50	60	1:1	30	−30
	60A	0.85% AMBIC	−50	60	1:1	30	−30
	60H10	10 mM HEPES	−50	60	1:1	30	−30
	60H70	70 mM HEPES	−50	60	1:1	30	−30
	70M	x	−50	70	1:1	35	−35
	100M	x	−50	100	1:1	50	−40
**Approach 3**	60–41	x	−50	60	1:4	48	−40
	100–21	x	−50	100	1:2	77	−45
**Controls**	Control (Unquenched)	x	x	x	x	x	x
	Media	x	x	x	x	x	x

## Supplementary Materials

The following are available online at http://www.mdpi.com/2218-1989/8/4/72/s1, Table S1: Summary of the main quenching and washing solvents applied for the metabolome analysis of different biological systems. (Sing x stands for no information available or provided in the research papers surveyed). Table S2: List of putatively identified metabolites in *C. reinhardtii* extracts across different applied quenching protocols (approach 1 and 3). Class 1 = Organic acids (non-fatty) and derivatives; 2 = Sugars/sugar alcohols and derivatives; 3 = Amino acid and derivatives; 4 = Nucleotides, nucleosides, nucleobases; 5 = Fatty acids/fatty alcohols and derivatives; 6 = Biogenic amines/Polyamine; 7 = Phosphates; 8 = Alkanes; 9 = Alcohols (other); 10 = Ketones and ethers; 11 = Others and 12 = Unknowns. Table S3: List of putatively identified metabolites in *C. reinhardtii* extracts across different applied quenching protocols (approach 2). Class 1 = Organic acids (non-fatty) and derivatives; 2 = Sugars/sugar alcohols and derivatives; 3 = Amino acid and derivatives; 4 = Nucleotides, nucleosides, nucleobases; 5 = Fatty acids/fatty alcohols and derivatives; 6 = Biogenic amines/Polyamine; 7 = Phosphates; 8 = Alkanes; 9 = Alcohols (other); 10 = Ketones and ethers; 11 = Others and 12 = Unknowns.
