# Supplementary material for: Quenching for Microalgal Metabolomics: A Case Study on the Unicellular Eukaryotic Green Alga Chlamydomonas reinhardtii
Source: Metabolites. 2018 Oct 31;8(4):72. doi: 10.3390/metabo8040072 (PMC6315863; doi:10.3390/metabo8040072)
Supplement: Supplementary file 1 [file metabolites-08-00072-s001.pdf]

Quenching for microalgal metabolomics: A case study on the unicellular eukaryotic green alga *Chlamydomonas reinhardtii*

Rahul Vijay Kapoore [1,2,*] and Seetharaman Vaidyanathan [1]

[1] Department of Chemical and Biological Engineering, ChELSI Institute, Advanced Biomanufacturing Centre, The University of Sheffield, Sheffield, S1 3JD, UK; S.Vaidyanathan@Sheffield.ac.uk
[2] Department of Biosciences, College of Science, Swansea University, Swansea, SA2 8PP, UK; R.V.Kapoore@swansea.ac.uk
* Correspondence: R.V.Kapoore@Swansea.ac.uk; Tel.: +44-(0)1792-51-3671

Table S1. Summary of the main quenching and washing solvents applied for the metabolome analysis of different biological systems. (Sing x stands for no information available or provided in the research papers surveyed).

Sample	***Culture volume (mL)***	***Quenching solvent (QS)***	***Quenching buffer***	***Temp.***	***Ratio (Sample to QS)***	***Washing solutions***	***Analytical Technique***	***Recommended QS***	***Ref.***
Penicillium chrysogenum	5	100% Methanol	x	(-40°C)	x	x	GC-MS and LC-ESI-MS/MS	40% Methanol	[1]
		60% Methanol		(-40°C)	1:5				
	10	40% Methanol		(-25°C)	1:1				
Pichia pastoris	2	60% Methanol	x	(-50°C)	1:7	Not required	GC-MS and NMR	Minor differences with all 4 QS, 60% Methanol + 0.11M AMBIC	[2]
		86% Methanol							
		60% Methanol +10 mM Tricine	10 mM Tricine						
		60% Methanol + 0.11M AMBIC	0.11 M AMBIC						
S. cerevisiae	1	100% Methanol	x		1:5	5 mL water	GC-ToF-MS	Fast filtration with water washing	[3]
S. cerevisiae	x	60% Methanol + 10 and/or 100 mM HEPES	10 and/or 100 mM HEPES	*(-40°C)*	1:5	*Not required*	GC-MS and LC-ESI-MS/MS	100% Methanol	*[4]*
		60% Methanol + 10 and/or100 mM AMBIC	10 and/or 100 mM AMBIC						
		60% Methanol + 10 and/or 100 mM Tricine	10 and/or 100 mM Tricine						
		40% Methanol	x						
		50% Methanol							
		60% Methanol							
		70% Methanol							
		80% Methanol							
Yeast (*Yarrowia lipolytica*)	7	60% Methanol	x	(-40°C)	1:4	50% Methanol	GC-MS	DMSO-saline	[5]
		Glycerol/NaCl (3:2)	NaCl	(-20°C)		Glycerol /NaCl (1:1)			
		20, 40 or 60% DMSO/NaCl	NaCl	(-4, -20 & -40°C)					
Yeast and Bacteria	x	40% Ethanol/NaCl	NaCl	(-20°C)	x	Not required	GC-MS	40% Methanol/0.8% NaCl	[6]
		60% Methanol	x	(-50°C)					
		Glycerol /NaCl (3:2)	NaCl	(-20°C)					
Yeast & Bacteria	5	Glycerol-water (3:2)	x	(-23°C)	1:5	x	GC-ToF-MS	Cold glycerol-saline	[7]
		Glycerol-saline (3:2)	NaCl						
Bacillus subtilis, C. glutamicum, E. coli, Gluconobacter oxydans, P. putida, Zymononas mobilis	x	60% Methanol + 10 mM HEPES	10 mM HEPES	(-58°C)	1:2	x	HPLC and IC-MS/MS	60% Methanol + 10 mM HEPES	[8]
C. glutamicum	5	60% Methanol + 10 mM HEPES	HEPES	(-58°C)	1:2	With wash	HPLC	Buffered methanol	[9]
		60% Methanol + 10 mM HEPES	HEPES			Without wash			
		60% Methanol	x			x			
		0.9% NaCl	x	(0.5°C)					
C. glutamicum		60% Methanol	x	(-50°C)	x	x	LC-MS		[10]
E. coli	5	LN2	x	(-40°C)	1:1	PBS	NMR	LN2	[11]
		60% Methanol							
E. coli	x	60% Methanol	x	(-50°C)	x	x	LC-MS		

		60% Methanol/Glycerol						60% Methanol/Glycerol	[12]
		60% Methanol + 10 mM HEPES	10 mM HEPES						
		60% Methanol + 70 mM HEPES	70 mM HEPES						
E. coli	1	60% Methanol	x	(-40°C)	1:5	Same as QS	GC-MS and LC-ESI-MS/MS	60% Methanol with differential method	[13]
		60% Methanol + 70 mM HEPES	70 mM HEPES						
		60% Methanol + 0.9% NaCl	0.9% NaCl						
		60% Methanol + 10 mM Tricine	10 mM Tricine						
E. coli	4	60% Methanol + 70 mM HEPES	70 mM HEPES	(-40°C)	1:5	x	Flow cytometry, ATP assay and OD based method	Neither	[14]
		60% Methanol + 40% glycerine/5.6% NaCl	NaCl						
		60% Methanol + Glutaraldehyde	Glutaraldehyde						
		60% Methanol + 5% Trehalose	Trehalose						
		60% Methanol + 0.5% Mannitol	Mannitol						
		40% glycerine/5.6% NaCl	NaCl						
		60% Methanol							
		100% Methanol							
L. bulgaricus	5	60% Methanol	x	(-20°C)	1:1	x	GC-MS & Flow cytometry	80% Methanol	[15]
		80% Methanol							
		80% Methanol/Glycerol							
CHO cells	x	60% Methanol + 70 mM HEPES	70 mM HEPES	(-20°C)	1:5	Not required	ATP Assay, Flow cytometry and LC-MS/MS	PBS (pH 7.4; 0.5°C)	[16]
		60% Methanol + 0.85% AMBIC	0.85% AMBIC	(-20°C)					
		PBS	x	(0.5°C)					
CHO cells	x	60% Methanol	x	(-40°C)	1:5	Not required	ATP assay, GC-ToF-MS, HPLC	60% Methanol + 0.85% AMBIC	[17]
		60% Methanol + 70 mM HEPES	70 mM HEPES						
		60% Methanol + 0.85% AMBIC	0.85% AMBIC						
		60% Methanol + 0.85% NaCl	0.85% NaCl						
Lactobacillus plantarum	x	60% Methanol	x	x	x	x	ATP assay and Fluorometry	60% Methanol + 0.85% AMBIC	[18]
		60% Methanol + 70 mM HEPES	70 mM HEPES						
		60% Methanol + 0.85% AMBIC	0.85% AMBIC						
		60% Methanol + 0.85% NaCl	0.85% NaCl						
Human macrophages	x	60% Methanol	x	(0.5°C)			GC-ToF-MS/HPLC-UV	0.9% NaCl	[19]
		40% Ethanol				0.9% NaCl			
		0.9% NaCl		4°C		0.9% NaCl			
Insect cells infected with virus	x	NaCl Based three QS	PF68	(-40°C)		Same as QS	HPLC and ATP Assay	NaCl + PF68 (Cell protectant) with 1 washing step	[20]
C. reinhardtii	1	70% methanol	x	(-70°C)	1:1	x	GC-ToF-MS	70% Methanol	[21]
C. reinhardtii	x	32.5% Methanol	x	(-25°C)	1:4	x	GC-ToF-MS	32.5% Methanol	[22]

Table S2. List of putatively identified metabolites in *C. reinhardtii* extracts across different applied quenching protocols (approach 1 & 3). Class 1 = Organic acids (non-fatty) and derivatives; 2 = Sugars/sugar alcohols and derivatives; 3 = Amino acid and derivatives; 4 = Nucleotides, nucleosides, nucleobases; 5 = Fatty acids/fatty alcohols and derivatives; 6 = Biogenic amines/Polyamine; 7 = Phosphates; 8 = Alkanes; 9 = Alcohols (other); 10 = Ketones and ethers; 11 = Others and 12 = Unknowns.

Met ID	***Metabolites***	***Class***
1	2-Piperidinecarboxylic acid	1
2	3-Indoleacetic acid	1
3	Aspartic acid	1
4	Eicosanoic acid	1
5	Erythronic acid	1
6	Fumaric acid	1
7	Glutaric acid	1
8	Glyceric acid	1
9	Glyoxylic acid	1
10	Gulonic acid	1
11	Iminodiacetic acid	1
12	Indole-2-carboxylic acid	1
13	Isobutanoic acid	1
14	Lactic acid	1
15	Lyxonic acid	1
16	Malic acid	1
17	Nicotinic acid	1
18	Orotic acid	1
19	Propanoic acid	1
20	Pyruvic acid	1
21	Shikimic acid	1
22	Threonic acid	1
23	Allose	2
24	Arabitol	2
25	Cellobiose	2
26	Cellotriose	2
27	Erythritol	2
28	Fructose	2
29	Galactitol	2
30	Galactonic acid	2
31	Galactopyranoside	2
32	Galactose	2
33	Galacturonic acid	2
34	Glucose	2
35	Glucuronic acid	2
36	Glycerol	2
37	Inositol	2
38	Kestose	2
39	Lactose	2
40	Laminaribiose	2
41	Lyxose	2
42	Maltotriose	2
43	Mannitol	2
44	Mannose	2
45	Ribitol	2
46	Ribose	2
47	Sedoheptulose	2
48	Sorbitol	2
49	Sorbose	2
50	Sucrose	2
51	Threitol	2
52	Trehalose	2
53	Xylitol	2
54	Xylose	2
55	Aminomalonic acid	3
56	Asparagine	3
57	Cysteamine	3
58	Cysteine	3
59	Glutamic acid	3
60	Glutamine	3
61	Glycine	3
62	Homoserine	3
63	Isoleucine	3
64	Leucine	3
65	Lysine	3
66	Norvaline	3
67	Ornithine	3
68	Phenylalanine	3
69	Proline	3
70	Prolyl-glycine	3
71	Purine	3
72	Pyroglutamic acid	3
73	Serine	3
74	Threonine	3
75	Tryptophan	3
76	Tyrosine	3
77	Valine	3
78	Adenine	4
79	Adenosine	4
80	Guanosine	4
81	Uracil	4
82	Docosahexaenoic acid	5
83	Docosanol	5
84	Dodecanoic acid	5
85	Dodecanol	5
86	Eicosanol	5
87	Heptadecanoic acid	5
88	Heptadecanol	5
89	Hexadecanoic acid	5
90	Hexadecanol	5
91	Hexadecenoic-acid	5
92	Nonadecanoic acid	5
93	Octadecadienoic acid	5
94	Octadecanoic acid	5
95	Octadecanol	5
96	Octadecatrienoic acid	5
97	Octadecenoic acid	5
98	Pentadecanoic acid	5
99	Pentadecanol	5
100	Tetradecanoic acid	5
101	Tridecanol	5
102	Ethanolamine	6
103	Phenethylamine	6

Table S2. Continued…

104	Putrescine	6
105	Sphingosine	6
106	Triethanolamine	6
107	Tryptamine	6
108	Dihydroxyacetone phosphate	7
109	Ethanolaminephosphate	7
110	Gluconic acid-6-phosphate	7
111	Glycerol-2-phosphate	7
112	Glycerol-3-phosphate	7
113	Glycolic acid-2-phosphate	7
114	Inositol-2-phosphate	7
115	Mannose-6-phosphate	7
116	myo-Inositol-1-phosphate	7
117	Ribose-5-phosphate	7
118	Ribulose-5-phosphate	7
119	Xylulose-5-phosphate	7
120	Decane	8
121	Docosane	8
122	Dodecane	8
123	Eicosane	8
124	Heneicosane	8
125	Heptadecane	8
126	Hexadecanal	8
127	Nonadecane	8
128	Octacosane	8
129	Octadecane	8
130	Pentacosane	8
131	Pentadecane	8
132	Tetradecane	8
133	Tricosane	8
134	Tridecane	8
135	Heneicosanol	9
136	Menthol	9
137	Phytol	9
138	Flavone	10
139	Butylamine	11
140	Carbodiimide	11
141	Cembrene	11
142	Cyclohexene	11
143	Hydantoin	11
144	Indole-3-acetaldehyde enol	11
145	Lumichrome	11
146	Naphthalene	11
147	Piceatannol	11
148	Quinazoline	11
149	Thymine	11
150	Tocopherol	11
151	Urea	11
152	UK1	12
153	UK10	12
154	UK11	12
155	UK12	12
156	UK13	12
157	UK14	12
158	UK15	12
159	UK16	12
160	UK17	12
161	UK18	12
162	UK19	12
163	UK2	12
164	UK20	12
165	UK21	12
166	UK22	12
167	UK23	12
168	UK24	12
169	UK25	12
170	UK26	12
171	UK27	12
172	UK28	12
173	UK29	12
174	UK3	12
175	UK30	12
176	UK31	12
177	UK32	12
178	UK33	12
179	UK34	12
180	UK35	12
181	UK36	12
182	UK37	12
183	UK38	12
184	UK39	12
185	UK4	12
186	UK40	12
187	UK41	12
188	UK42	12
189	UK43	12
190	UK44	12
191	UK45	12
192	UK5	12
193	UK5	12
194	UK6	12
195	UK7	12
196	UK8	12
197	UK9	12

Table S3. List of putatively identified metabolites in *C. reinhardtii* extracts across different applied quenching protocols (approach 2). Class 1 = Organic acids (non-fatty) and derivatives; 2 = Sugars/sugar alcohols and derivatives; 3 = Amino acid and derivatives; 4 = Nucleotides, nucleosides, nucleobases; 5 = Fatty acids/fatty alcohols and derivatives; 6 = Biogenic amines/Polyamine; 7 = Phosphates; 8 = Alkanes; 9 = Alcohols (other); 10 = Ketones and ethers; 11 = Others and 12 = Unknowns.

Met ID	*Metabolites*	*Class*
1	2-Piperidinecarboxylic acid	1
2	Adipic acid	1
3	Aspartic acid	1
4	Erythronic acid	1
5	Fumaric acid	1
6	Glyoxylic acid	1
7	Gulonic acid	1
8	Iminodiacetic acid	1
9	Indole-2-carboxylic acid	1
10	Isobutanoic acid	1
11	Lactic acid	1
12	Lyxonic acid	1
13	Malic acid	1
14	Nicotinic acid	1
15	Shikimic acid	1
16	Threonic acid	1
17	Arabitol	2
18	Cellobiose	2
19	Erythritol	2
20	Erythrulose	2
21	Fructose	2
22	Galactitol	2
23	Galactopyranoside	2
24	Glucose	2
25	Glycerol	2
26	Inositol	2
27	Kestose	2
28	Lyxose	2
29	Mannitol	2
30	Mannose	2
31	Psicose	2
32	Ribitol	2
33	Ribose	2
34	Sorbitol	2
35	Sorbose	2
36	Sucrose	2
37	Threitol	2
38	Threose	2
39	Xylitol	2
40	Xylose	2
41	Alanine	3
42	Aminomalonic acid	3
43	Cysteamine	3
44	Cysteine	3
45	Glutamic acid	3
46	Glutamine	3
47	Glycine	3
48	Homoserine	3
49	Isoleucine	3
50	Leucine	3
51	Lysine	3
52	Phenylalanine	3
53	Proline	3
54	Prolyl-glycine	3
55	Pyroglutamic acid	3
56	Serine	3
57	Threonine	3
58	Tryptophan	3
59	Tyrosine	3
60	Valine	3
61	Adenosine	4
62	Guanosine	4
63	Uracil	4
64	Dodecanoic acid	5
65	Dodecanol	5
66	Eicosanol	5
67	Heptadecanoic acid	5
68	Heptadecanol	5
69	Hexadecanoic acid	5
70	Hexadecanol	5
71	Hexadecenoic-acid	5
72	Octadecadienoic acid	5
73	Octadecanoic acid	5
74	Octadecanol	5
75	Octadecatrienoic acid	5
76	Octadecenoic acid	5
77	Pentadecanoic acid	5
78	Pentadecanol	5
79	Tetradecanoic acid	5
80	Tridecanol	5
81	Ethanolamine	6
82	Phenethylamine	6
83	Putrescine	6
84	Sphingosine	6
85	Triethanolamine	6
86	Tryptamine	6
87	Erythrose-4-phosphate	7

Table S3. Continued…

88	Ethanolaminephosphate	7
89	Gluconic acid-6-phosphate	7
90	Glucose-6-phosphate	7
91	Glycerol-2-phosphate	7
92	Glycerol-3-phosphate	7
93	Glycolic acid-2-phosphate	7
94	Inositol-1-phosphate	7
95	Mannose-6-phosphate	7
96	myo-Inositol-1-phosphate	7
97	Xylulose-5-phosphate	7
98	Decane	8
99	Docosane	8
100	Eicosane	8
101	Heneicosane	8
102	Heptadecane	8
103	Hexadecanal	8
104	Nonadecane	8
105	Octadecane	8
106	Pentacosane	8
107	Pentadecane	8
108	Tetradecane	8
109	Tricosane	8
110	Tridecane	8
111	Ampelopsin	9
112	Heneicosanol	9
113	Menthol	9
114	Phytol	9
115	Flavone	10
116	Butylamine	11
117	Hydantoic acid	11
118	Lumichrome	11
119	Nicotinamide	11
120	Phenylpyruvic acid	11
121	Thiophene	11
122	Tocopherol	11
123	Urea	11
124	UK1	12
125	UK10	12
126	UK11	12
127	UK12	12
128	UK13	12
129	UK14	12
130	UK15	12
131	UK16	12
132	UK17	12
133	UK18	12
134	UK19	12
135	UK2	12
136	UK20	12
137	UK21	12
138	UK22	12
139	UK23	12
140	UK24	12
141	UK25	12
142	UK26	12
143	UK27	12
144	UK28	12
145	UK29	12
146	UK3	12
147	UK30	12
148	UK31	12
149	UK32	12
150	UK33	12
151	UK34	12
152	UK35	12
153	UK36	12
154	UK37	12
155	UK38	12
156	UK39	12
157	UK4	12
158	UK40	12
159	UK41	12
160	UK42	12
161	UK5	12
162	UK6	12
163	UK7	12
164	UK8	12
165	UK9	12

References

1. de Jonge, L.P.; Douma, R.D.; Heijnen, J.J.; van Gulik, W.M. Optimization of cold methanol quenching for quantitative metabolomics of Penicillium chrysogenum. *Metabolomics* **2012**, *8*, 727-735.
2. Tredwell, G.D.; Edwards-Jones, B.; Leak, D.J.; Bundy, J.G. The development of metabolomic sampling procedures for Pichia pastoris, and baseline metabolome data. *PloS one* **2011**, *6*, e16286.
3. Kim, S.; Lee, D.Y.; Wohlgemuth, G.; Park, H.S.; Fiehn, O.; Kim, K.H. Evaluation and optimization of metabolome sample preparation methods for Saccharomyces cerevisiae. *Analytical chemistry* **2013**, *85*, 2169-2176.
4. Canelas, A.B.; Ras, C.; ten Pierick, A.; van Dam, J.C.; Heijnen, J.J.; Van Gulik, W.M. Leakage-free rapid quenching technique for yeast metabolomics. *Metabolomics* **2008**, *4*, 226-239.
5. Zhao, C.; Nambou, K.; Wei, L.; Chen, J.; Imanaka, T.; Hua, Q. Evaluation of metabolome sample preparation methods regarding leakage reduction for the oleaginous yeast< i> Yarrowia lipolytica</i>. *Biochemical Engineering Journal* **2014**, *82*, 63-70.
6. Spura, J.; Christian Reimer, L.; Wieloch, P.; Schreiber, K.; Buchinger, S.; Schomburg, D. A method for enzyme quenching in microbial metabolome analysis successfully applied to gram-positive and gram-negative bacteria and yeast. *Analytical biochemistry* **2009**, *394*, 192-201.
7. Villas-Bôas, S.G.; Bruheim, P. Cold glycerol–saline: The promising quenching solution for accurate intracellular metabolite analysis of microbial cells. *Analytical biochemistry* **2007**, *370*, 87-97.
8. Bolten, C.J.; Kiefer, P.; Letisse, F.; Portais, J.-C.; Wittmann, C. Sampling for metabolome analysis of microorganisms. *Analytical chemistry* **2007**, *79*, 3843-3849.
9. Wittmann, C.; Krömer, J.O.; Kiefer, P.; Binz, T.; Heinzle, E. Impact of the cold shock phenomenon on quantification of intracellular metabolites in bacteria. *Analytical biochemistry* **2004**, *327*, 135-139.
10. Wellerdiek, M.; Winterhoff, D.; Reule, W.; Brandner, J.; Oldiges, M. Metabolic quenching of Corynebacterium glutamicum: efficiency of methods and impact of cold shock. *Bioprocess and biosystems engineering* **2009**, *32*, 581-592.
11. Bertini, I.; Hu, X.; Luchinat, C. Global metabolomics characterization of bacteria: pre-analytical treatments and profiling. *Metabolomics*, 1-9.
12. Link, H.; Anselment, B.; Weuster-Botz, D. Leakage of adenylates during cold methanol/glycerol quenching of Escherichia coli. *Metabolomics* **2008**, *4*, 240-247.
13. Taymaz-Nikerel, H.; De Mey, M.; Ras, C.; ten Pierick, A.; Seifar, R.M.; Van Dam, J.C.; Heijnen, J.J.; van Gulik, W.M. Development and application of a differential method for reliable metabolome analysis in< i> Escherichia coli</i>. *Analytical Biochemistry* **2009**, *386*, 9-19.
14. Schädel, F.; David, F.; Franco-Lara, E. Evaluation of cell damage caused by cold sampling and quenching for metabolome analysis. *Applied microbiology and biotechnology* **2011**, *92*, 1261-1274.
15. Chen, M.-m.; Li, A.-l.; Sun, M.-c.; Feng, Z.; Meng, X.-c.; Wang, Y. Optimization of the quenching method for metabolomics analysis of Lactobacillus bulgaricus. *Journal of Zhejiang University SCIENCE B* **2014**, *15*, 333-342.
16. Kronthaler, J.; Gstraunthaler, G.; Heel, C. Optimizing high-throughput metabolomic biomarker screening: a study of quenching solutions to freeze intracellular metabolism in CHO cells. *Omics: a journal of integrative biology* **2012**, *16*, 90-97.
17. Sellick, C.A.; Hansen, R.; Maqsood, A.R.; Dunn, W.B.; Stephens, G.M.; Goodacre, R.; Dickson, A.J. Effective quenching processes for physiologically valid metabolite profiling of suspension cultured mammalian cells. *Analytical chemistry* **2008**, *81*, 174-183.
18. Faijes, M.; Mars, A.E.; Smid, E.J. Comparison of quenching and extraction methodologies for metabolome analysis of Lactobacillus plantarum. *Microbial cell factories* **2007**, *6*, 27.
19. Cheng, J.; Che, N.; Li, H.; Ma, K.; Wu, S.; Fang, J.; Gao, R.; Liu, J.; Yan, X.; Li, C. Extraction, derivatization, and determination of metabolome in human macrophages. *Journal of separation science* **2013**, *36*, 1418-1428.
20. Tran, T.T.; Dietmair, S.; Chan, L.C.; Huynh, H.T.; Nielsen, L.K.; Reid, S. Development of quenching and washing protocols for quantitative intracellular metabolite analysis of uninfected and baculovirus-infected insect cells. *Methods* **2012**, *56*, 396-407.

21. Lee, D.Y.; Fiehn, O. High quality metabolomic data for Chlamydomonas reinhardtii. *Plant methods* **2008**, *4*, 7.
22. Bölling, C.; Fiehn, O. Metabolite profiling of Chlamydomonas reinhardtii under nutrient deprivation. *Plant Physiology* **2005**, *139*, 1995-2005.
